# Optimization of Drug Delivery Systems for Intraperitoneal Therapy to Extend the Residence Time of the Chemotherapeutic Agent

**DOI:** 10.1155/2013/720858

**Published:** 2013-03-25

**Authors:** L. De Smet, W. Ceelen, J. P. Remon, C. Vervaet

**Affiliations:** ^1^Laboratory of Pharmaceutical Technology, Ghent University, Harelbekestraat 72, 9000 Ghent, Belgium; ^2^Laboratory of Experimental Surgery, Ghent University Hospital, De Pintelaan 185, 9000 Ghent, Belgium

## Abstract

Intraperitoneal (IP) chemotherapy is an effective way of treating peritoneal carcinomatosis of colorectal origin after complete cytoreduction. Although IP therapy has been already performed for many years, no standardized treatment design has been developed in terms of schedule, residence time, drug, or carrier solution. Because of the fast clearance of the conventional intravenous (IV) drug delivery systems used for IP therapy, a lot of research is performed to optimize IP drug delivery and extend the residence time of the cytotoxic agent in the peritoneal cavity. This paper reviews the recent advances made in drug delivery systems for IP chemotherapy, discussing the use of microparticles, nanoparticles, liposomes, micelles, implants, and injectable depots for IP delivery.

## 1. Introduction

Colorectal cancer is the third most common cancer and ranks as the fourth most common cancer-related mortality [1]. A major component of treatment failure is cancer dissemination within the abdominal and pelvic spaces as a local recurrence of the primary cancer or as peritoneal carcinomatosis (PC). It is estimated that about 40% of the patients with colorectal cancer will develop PC during the evolution of the disease [2] as a result of the growth of the primary tumor through the serosal lining of the bowel lumen, thereby allowing the exfoliation and shedding of malignant cells intraperitoneally. Manipulation during the surgical procedure may also release tumor cells within the peritoneal cavity [2].

PC from colorectal cancer was traditionally regarded as a terminal condition. The terminal nature of this disease has been demonstrated in a large clinical trial, the French multicentric EVOCAPE-1 trial, which prospectively followed up 370 patients with PC of different primary cancers from diagnosis till death. The mean survival of 118 patients with PC from colorectal cancer was 6.0 months [3].

For over two decades, adjuvant intravenous (IV) therapy with 5-fluorouracil (5-FU) has improved the therapeutic outcome after cytoreduction [4]. After the introduction of oxaliplatin in combination with 5-FU or leucovorin, the effectiveness of systemic treatment of PC was improved [5, 6]. A combination of these agents in randomized clinical trials in patients with metastatic colorectal cancer increased the median survival up to 20 months [7, 8]. This combination IV therapy is the current standard of care for adjuvant therapy of resected colon cancer.

Since the 1980s, different treatment options for patients with isolated PC were proposed based on the hypothesis that PC is a local-regional disease which would benefit from a local therapeutic approach [9]. The rationale of using local intraperitoneal (IP) therapy for the treatment of PC is based on the pharmacokinetic advantages, such as high local concentration with a longer half-life of the drug in the peritoneal cavity, which improves the interaction of the cytotoxic agents with the cancer cells.

Over the last decades, some researchers focused on improving the drug delivery systems for IP therapy. Research towards IP drug delivery has focused on the means to obtain higher drug concentrations at the site of the disease in combination with a reduction of the toxicity to healthy tissues. This paper reviews the different strategies that have been developed to control drug release in combination with a low systemic toxicity and an extended residence time of the cytotoxic drug in the peritoneal cavity [10]. Micro- and nanoparticles, liposomes, micelles, implants, and injectable depots are discussed. 

## 2. Intraperitoneal Chemotherapy

The current treatment protocols for peritoneal carcinomatosis are based on a combination of cytoreductive surgery (CRS) and perioperative intraperitoneal chemotherapy.

The first step is to eliminate all macroscopic disease by CRS since the size of the residual tumor nodules is the major prognostic indicator for the outcome of the disease. CRS is considered complete when no visible evidence of cancer remains (CC-0), or when only small residual nodules with a size less than 2.5 mm remain (CC-1), as this small size ensures full drug penetration into the tumor during subsequent intraperitoneal chemotherapy. CRS with residual tumors with a diameter >2.5 mm (CC-2 and CC-3) is considered to be incomplete, and these patients have a low survival rate [11, 12].

A second essential part of the current management of PC is the perioperative intraperitoneal chemotherapy which targets the residual microscopic disease. IP treatment of patients with PC was first reported by Spratt et al., who used triethylenethiophosphoramide in a patient with pseudomyxoma peritonei [9], and by Speyer and Myers, who administered 5-FU and methotrexate intraperitoneally under normothermic conditions to 16 patients with PC [13]. In 1988, Koga et al. reported intraperitoneal chemotherapy in 23 patients with PC from gastric cancer origin [14].

The adjuvant perioperative intraperitoneal chemotherapy can be applied directly after CRS under normothermic or hyperthermic conditions (i.e., (hyperthermic) intraperitoneal chemotherapy or (H)IPEC) as a part of the surgical procedure or can be started on the first postoperative day and continued for several (usually five) days (i.e., early postoperative intraperitoneal chemotherapy or EPIC). No randomized trials have been published comparing the survival outcomes between EPIC and HIPEC [15]. EPIC has been associated with a higher risk of complications after CRS, possibly due to the prolonged contact between the chemotherapeuticum and damaged tissues at the operated surfaces [16]. Other disadvantages of EPIC are an uneven distribution of the chemotherapeutic fluid in the abdomen and causing less comfort for the patients (e.g., nausea and impaired mobility) linked to the chemotherapeutic fluid present in the abdomen [17]. As the main drawback of HIPEC is the necessity to perform this technique in the operating room, requiring specialized equipment and an experienced perfusionist, HIPEC is the preferred technique. When performing the open HIPEC technique, the abdomen is covered with a plastic sheet, aerosolized droplets for the IP heated chemotherapy are aspirated and the surgeon who is manipulating the viscera is wearing special sterile gloves; each of these a precaution to minimize the risk of exposure of the operating room personnel to the chemotherapeuticum [18]. In most cases HIPEC is performed under hyperthermic conditions (41-42°C) as hyperthermia is claimed to enhance the effect of different cytotoxic agents (e.g., mitocyn C, doxorubicin, cisplatin, and oxaliplatin) [19]. However, some studies suggest that there is no synergistic effect between the cytotoxic agent and hyperthermia, and no differences were observed when performing IP at normothermic or hyperthermic conditions [15, 20].

Promising results when combining CRS and perioperative chemotherapy were reported in several studies. HIPEC with mitomycin C followed by systemic chemotherapy resulted in a 45% 5-year survival in colorectal patients receiving complete cytoreduction [21]. In a second study the 5-year survival among 1290 patients with PC from different primary origins receiving CRS and perioperative chemotherapy was 37% [12].

## 3. Drug Selection for IP Therapy

Although IP therapy is already conducted for many years, no standard treatment in terms of schedule, residence time, drug, or carrier solution has been established. In the case of IP therapy, the cytotoxic agent must be able to penetrate the peritoneal surface and the tumor nodules effectively, in combination with its ability to eradicate microscopic residual disease within the peritoneal fluid. During IP therapy, drug delivery to peritoneal tumors is twofold. The primary route is drug diffusion through the tumor interstitium, while the second route is the recirculation of the drug absorbed from the peritoneal cavity. However, it is evident that the latter route is of minor importance as efficient and safe IP therapy requires systemic drug levels as low as possible in order to minimize systemic side effects caused by the cytotoxic agent.

One of the major challenges of IP therapy is to maintain a high local drug concentration within the peritoneal cavity to provide a sufficient concentration gradient as driving force for drug diffusion into the tumor. The residence time of small molecular weight drugs in the peritoneal cavity may not be sufficient because they are quickly absorbed through the peritoneal capillaries into the systemic circulation [22]. Therefore, the drug delivery system used for IP therapy is an important factor to deliver drugs efficiently to the target tissue. In addition, different parameters must be considered to select the ideal chemotherapeutic agent for IP therapy and to maximize its efficacy: cavity-to-plasma AUC ratio, systemic absorption, depth of tumor penetration, and intrinsic activity of the agent against the primary tumor type. In general, water insoluble molecules with a high molecular weight and a high cavity-to-plasma AUC ratio remain longer in the peritoneal cavity and are thus preferred for IP treatment (Table 1) [23, 24]. One important consideration is that a high cavity-to-plasma AUC ratio does not automatically confer a higher efficacy, since penetration of the chemotherapeutic agent into the tumor might be limited.

## 4. Drug Delivery Systems for IP Therapy

Inadequate drug delivery to solid tumors is a major cause of IP treatment failure. Today there are no chemotherapeutic formulations specifically approved for IP therapy, and current IP chemotherapy in patients relies on the off-label use of products developed for IV applications. These formulations designed for IV therapy often suffer from a rapid clearance from the peritoneal cavity, fast absorption through the lymphatics, no tumor selectivity, and local and systemic toxicity caused by the high blood concentrations after systemic absorption. These issues, which reduce the efficacy of IP therapy, dramatically limit the use of conventional IV chemotherapeutic formulations for IP use [25]. As a result, a number of drug delivery strategies (which are reviewed in the remainder of this paper) have been investigated to extend the residence time of the chemotherapeutic agent and to optimize IP therapy (Table 2).

### 4.1. Microspheres

Microspheres (>1 *μ*m) can be designed to release the drug gradually over time using a wide variety of biodegradable and biocompatible polymeric substances. The size of the microspheres is the most important factor that influences residence time in the peritoneum, since microspheres smaller than 8 *μ*m may disappear from the peritoneal cavity through the lymphatic capillaries [26]. 

Microspheres for IP drug delivery are often based on biodegradable aliphatic polyesters of hydroxyl acids such as poly(lactic-*co*-glycolic)acid (PLGA) and related polymers. PLGA was, for example, used to incorporate cisplatin (*cis*-dichloro diammmine-platinum(II)) in microspheres. Cisplatin is a hydrophilic molecule which is, in solution, rapidly absorbed from the peritoneal cavity by the capillaries and transferred to the systemic circulation. Therefore biodegradable anticancer drug-loaded microspheres were developed which showed a long retention and sustained release of the drug. Cisplatin was released from the PLGA matrix by diffusion until 14 days after IP administration. During this period the particles were retained in the abdominal cavity for a long period and gradually absorbed, which reduced the systemic side effects [27]. Another drug delivery system using PLGA is paclitaxel-loaded microparticles [28]. This system consists of two types of paclitaxel-loaded microparticles with different drug release rates obtained by the lactide/glycolide (L/G) ratio. First, a burst release was observed (L/G 50 : 50), followed by a sustained drug release (L/G 75 : 25). As paclitaxel is a promising molecule for IP treatment, paclitaxel-containing microsphere formulations based on other polymers were also developed. Paclimer was made by incorporating paclitaxel in a biodegradable poly-phosphoester polymer matrix, to form microspheres with a mean particle size of 53 *μ*m. These microparticles resulted both in vitro and in vivo (after IP administration in a phase I study) in a sustained release of paclitaxel over a period of 8 weeks [29]. The triblock poly(*ε*-caprolactone)-poly(ethyleneglycol)-poly(*ε*-caprolactone) (PCL-PEG-PCL) copolymer was synthesized to prepare camptothecin-loaded microspheres. These microspheres were developed to protect camptothecin from hydrolysis, thus enhancing its treatment efficacy towards colorectal peritoneal carcinomatosis [30].

### 4.2. Nanoparticles

Although microspheres have a longer retention time in the peritoneal cavity, they can also induce inflammatory reactions and peritoneal adhesions [31]. Because of these issues, the benefit-to-risk ratio should be taken into account when deciding between microparticles and nanoparticles. Kohane et al. showed that nanoparticles and microparticles formulated with lower molecular weight polymers had a much lower incidence of peritoneal adhesions and were safer to use [32]. Another advantage of nanoparticles is that they can bypass drug efflux pumps, thus evading multidrug resistance and achieving significantly higher drug accumulation in the cells compared to IP therapy with the unformulated free drugs [33, 34]. Although conventional nanoparticles are rapidly cleared from the abdominal cavity due to their size, nanoparticles which respond to a wide array of stimuli such as pH, temperature, light, and ultrasound are being investigated. For IP therapy, paclitaxel-loaded pH responsive nanoparticles were developed which were designed to deliver paclitaxel intracellularly after endocytosis. In this formulation paclitaxel is encapsulated in an acrylate-based polymer with a pH-responsive 2,4,6-trimethoxybenzaldehyde protective group. These nanoparticles react to an endosomal pH (pH ≤5) and increase in volume to release their drug load. When these nanoparticles are IP injected in a mice tumor model, they remain in the peritoneal cavity for 7 days [35].

### 4.3. Liposomes

Liposomes have been widely studied as potential carriers for hydrophilic and hydrophobic drugs and diagnostic agents. Due to their small size (100–1000 nm), liposomes have a fast clearance from the abdominal cavity. As such it is important to change some of the liposomes characteristics such as lipid composition, surface properties, and charge in order to increase their retention time in the abdominal cavity. Hirano et al. (1985) described that the charge of the liposomes is a predictive factor for the retention time [22]. When the liposomes have a negative charge, they were rapidly absorbed from the peritoneal cavity, while positively charged liposomes had a slower absorption rate. This might be attributed to electrostatic interaction between the positively charged liposomes and the negative surface of the peritoneal mesothelium, in combination with a low uptake of positive liposomes by peritoneal macrophages [36]. Changing the type of phospholipid (the main building block of liposomes) has no effect on retention time in the abdominal cavity [37], whereas the incorporation of polyethylenglycol (PEG) in the phospholipid membrane showed a 30% higher peritoneal retention by the avoidance of the macrophages present in the peritoneal cavity, compared to the same nonpegylated liposomes [36]. 

### 4.4. Micelles

Taxol, a micellar paclitaxel formulation using Cremophor EL (i.e., a polyethoxylated castor oil surfactant), is used for IP treatment of ovarian cancer. Taxol showed a longer residence time in the abdominal cavity compared to free unformulated paclitaxel (40.7 ± 13.8 h versus 7.3 ± 2.8 h), which indicated that encapsulation is an effective way to extend the residence time of a drug in the abdominal cavity [38]. However due to the surfactant (Cremophor EL) included in the formulation, Taxol is not well tolerated as hypersensitivity reactions and neurotoxicity are reported [39]. IP chemotherapy using a nanocrystalline paclitaxel formulation stabilized by Pluronic F127 (i.e., polyethylene oxide-polypropylene oxide (PEO-PPO) block copolymer) showed a faster recovery of the animals compared to treatment with Taxol, while the cytotoxicity and antitumor efficacy was similar [40]. Several studies showed that the carrier plays an important factor in the distribution and clearance of the drugs after IP administration [38, 41]. 4.2% Cremophor EL and 1.5% polysorbate-80 were used to solubilize paclitaxel and docetaxel, respectively. In a rat model, the absorption rate of these taxanes was influenced by the solubilizer in the system. While the AUC_ip_ of paclitaxel doubled in comparison to docetaxel, when conventional vehicles were used, dissolution of docetaxel in 4.2% Cremophor EL or 7.5% polysorbate-80 increased its retention time and yielded similar AUC_ip_ values compared to paclitaxel [42].

### 4.5. Implants and Injectable Depots

Implantable and injectable depots have been investigated for localized and sustained delivery of anticancer agents [43]. Those systems may be the most promising approach for the IP treatment of PC. The rationale of using hydrogels for IP therapy is dual as a hydrogel will retain the drugs within the peritoneum and will also protect against peritoneal adhesions [44]. A major drawback for hydrogels is achieving homogeneous distribution of the drug in the peritoneal cavity. Also the viscosity of the injectable hydrogels can cause problems. Low viscous systems may fail to provide a delayed drug release profile, while high viscous systems may be difficult to administer [43]. Because of these problems, thermosensitive hydrogels are developed. These gels are free-flowing at room temperature and form a nonflowing gel at body temperature, serving as an in situ drug depot. Therefore the formulation can be easily mixed with the drugs and injected by a syringe as a fluid, while forming a viscous deposition at the target location [45]. This gelation behavior is primarily due to the formation of self-associated micelles via hydrophobic interactions. Above the lower critical solution temperature, these micelles are closely packed together resulting in micellar aggregation and a change of rheological properties [46]. Gong et al. reported that a hydrogel system based on a biodegradable poly(ethylene glycol)-poly(*ε*-caprolactone)-poly(ethylene glycol) (PEG-PCL-PEG, PECE) triblock copolymer could be used as a delivery vector of chemotherapeutic drugs for IP infusion chemotherapy. The system was loaded with 5-FU [47] and doxorubicin [48]. When the system was infused intraperitoneally in mice, the formed gel phase guaranteed a delayed drug release up to 48 hours after injection.

Implantable systems were developed for IP treatment of ovarian cancer. This implant is composed of paclitaxel-loaded poly-D,L-lactide, and poly(lactide)-block-poly(ethylene glycol) (PLA-b-PEG) particles dispersed throughout a chitosan egg-phosphatidylcholine matrix. This formulation provided a sustained and localized release of 1% PTX per day in mice over a period of 3 months [49, 50]. The implants have a higher efficacy and are less toxic and more biocompatible than Taxol. Although the implantable systems have promising results, the biggest issue for using those systems is the need for surgical expertise to implant the system.

### 4.6. Carrier Solutions

A simple approach to improve IP administration of chemotherapeutic agents using conventional formulations is varying the medium in which the drug is dissolved or suspended. The volume of the fluid in which the chemotherapeutic agent is dissolved plays an important role towards drug distribution in the peritoneal cavity. The ideal carrier solution for IP chemotherapy should expose all cancerous surfaces or residual tumor cells to high levels of cytotoxic agents for as long as possible and ensure a uniform distribution of the drug in the abdominal cavity. Small volumes of fluid do not flow freely in the peritoneum, even with multiple position changes of the patients [51], while large volumes (>2 L/m^2^ body surface area) which cause moderate abdominal distention result in more uniform intraperitoneal drug distribution. Hence maintaining a high volume of the intraperitoneal liquid would improve the effectiveness of the treatment as the choice of the solution, in which the drug is administered, plays an important role in the distribution of the drugs [52, 53]. Current techniques for IP chemotherapy administration mainly use isotonic electrolyte solutions (e.g., 5% glucose and 0.9% sodium chloride). However isotonic salt and dextrose solutions are rapidly absorbed due to their low molecular weight [52–54] which decreases the distribution of the drug in the peritoneal cavity. Hypotonic solutions showed promising in vitro results as they increased cytotoxicity and accumulation of cisplatin in tumor cells [55]. However, the clinical results were negative as bleeding and thrombocytopenia occurred, while no pharmacokinetic advantages were observed [56, 57]. When hypertonic solutions were used, a prolonged retention time of the intraperitoneal volume was achieved, but their main disadvantage is the dilution of the intraperitoneal drug due to fluid shift to the peritoneal cavity [52]. 4% icodextrin, a colloid osmotic agent of the *α*-1,4 linked glucose polymer, has been successfully used to prolong retention of intraperitoneal chemotherapy; the solution can remain for 3 to 4 days in the peritoneum [58]. Another isoosmolar solution, with a potentially long intraperitoneal dwell time, is 6% hydroxyethyl starch (hetastarch) which has been successfully used to prolong intraperitoneal retention of gemcitabine and paclitaxel in animal studies [52, 59]. Also the use of HAES-steril, a starch-based carrier solution, reduced the clearance of the chemotherapeutic solutions from the peritoneal cavity compared to physiological saline solution [60]. 

## 5. Conclusion

Cytoreductive surgery followed by intraoperative intraperitoneal chemotherapy has proven to be a very effective treatment for patients with peritoneal carcinomatosis of colorectal origin. 

For the moment, there are no chemotherapeutic agents specifically designed or approved for IP therapy. This paper indicates the important role of the different drug delivering systems to deliver the cytotoxic agents more adequately. The requirements for a good drug delivery systems for IP therapy are that high locoregional drug concentrations are maintained for a longer period in combination with a lower nonspecific toxicity. If these drug delivery systems can be combined with high molecular weight drugs with a high peritoneal cavity-to-plasma AUC ratio, this could result in a more effective way of treating PC. 

## Figures and Tables

**Table 1:** Water solubility, the partition coefficient (log *P*), and peritoneal to plasma drug area under the curve (AUC) ratio of intraperitoneal chemotherapeutic agents.

Chemotherapeutic agent	Molecular weight	Water solubility	log *P *	Peritoneal to plasma AUC ratio
Cisplatin	300	Good	−2.19	12
Carboplatin	371	Good	1.06	10–18
Oxaliplatin	397	Good	1.73	16
Paclitaxel	854	Poor	3.54	1000
Docetaxel	862	Poor	2.92	181
5-Fluorouracil	130	Sparing	-0.89	367
Doxorubicin	544	Poor	1.27	474

**Table 2:** Advantages and disadvantages of the drug delivery systems investigated for IP therapy.

Drug delivery system	Advantages	Disadvantages
Microspheres	Prolonged retention time	Limited tumor penetration Risk of peritoneal adhesions
Nanoparticles	Small size passive targeting Avoiding MDR Lower incidence of peritoneal adhesions	Rapid clearance out of the abdominal cavity
Liposomes	Similar to nanoparticles Active targeting by varying parameters	Similar to nanoparticles
Micelles	Prolonged retention time	Increasing systemic toxicity
Injectable systems	Prolonged retention time Localized and sustained drug delivery Lower systemic toxicity Prevention against peritoneal adhesion	Viscosity issues
Implantable systems	Similar to injectable systems	Invasive Surgical expertise
